# Efficacy and Safety of OnabotulinumtoxinA in Patients with Neurogenic Detrusor Overactivity: A Systematic Review and Meta-Analysis of Randomized Controlled Trials

**DOI:** 10.1371/journal.pone.0159307

**Published:** 2016-07-27

**Authors:** Tao Cheng, Wei-bing Shuang, Dong-dong Jia, Min Zhang, Xu-nan Tong, Wei-dong Yang, Xu-ming Jia, Shuo Li

**Affiliations:** 1 The First Clinical Medical College of Shanxi Medical University, Taiyuan, Shanxi, China; 2 Zhejiang Cancer Hospital, Hangzhou, Zhejiang, China; 3 Affiliated Hospital of Hebei University, Baoding, Hebei, China; 4 ShanXi Hospital of Integrated Traditional and Western Medicine, Taiyuan, Shanxi, China; 5 Department of Urology, First Hospital of Shanxi Medical University, Taiyuan, Shanxi, China; Cardiff University, UNITED KINGDOM

## Abstract

**Background:**

Neurogenic detrusor overactivity (NDO) affects the quality of life (QoL) of millions of individuals worldwide. The purpose of this study was to assess the efficacy and safety of onabotulinumtoxinA in patients with NDO using a network meta-analytic approach, which can also quantify and compare the efficacy of onabotulinumtoxinA across different dosages.

**Methods:**

PubMed, EMBASE, and the Controlled Trials Register were searched to identify randomized controlled trials comparing onabotulinumtoxinA to a control for NDO in adult patients. The primary outcome was the mean number of urinary incontinence (UI) episodes per week. Urodynamic parameters included the maximum cystometric capacity (MCC) and the maximum detrusor pressure (MDP). The safety of onabotulinumtoxinA was determined by the incidence of various frequent adverse events (AEs). Two authors extracted data independently, and the statistical analyses were performed using RevMan 5.1.0 software.

**Results:**

A total of 1,915 patients from six randomized controlled trials were included in this meta-analysis. The onabotulinumtoxinA-treated groups had a significantly decreased mean number of urinary incontinence episodes per week (at week 6) (onabotulinumtoxinA200U: MD: -10.72, 95% CI: -13.4 to -8.04, P<0.00001; 300 U: MD: -11.42, 95% CI: -13.91 to -8.93, P<0.00001), MDP (200 U: MD: -33.46, 95% CI: -39.74 to -27.18, P<0.00001; 300 U: MD: -31.72, 95% CI: -37.69 to -25.75, P<0.00001), and greater increased MCC (200 U: MD: 141.30, 95% CI: 121.28 to 161.32, P<0.00001; 300 U: MD: 151.39, 95% CI: 130.43 to 172.34, P<0.00001) compared to the placebo-treated groups. However, there were no significant differences between the onabotulinumtoxinA-treated groups for the number of weekly UI episodes at 6 weeks (MD: 0.08, 95% CI: -2.57 to 2.73, P = 0.95). Similarly, we also observed that there were no significant differences in MCC (MD: -9.97, 95% CI: -33.15 to 13.20, P = 0.40) and MDP (MD: -1.86, 95% CI: -8.09 to 4.37, P = 0.56). Considering the AEs, the onabotulinumtoxinA-treated groups were often associated with more complications, including urinary tract infections (UTIs) (RR: 1.47, 95% CI: 1.29 to 1.67, P<0.00001), urinary retention (RR: 5.58, 95% CI: 3.53 to 8.83, P<0.00001), hematuria (RR: 1.70, 95% CI: 1.01 to 2.85, P = 0.05), and muscle weakness (RR: 2.59, 95% CI: 1.36 to 4.91, P = 0.004).

**Conclusions:**

OnabotulinumtoxinA can significantly reduce the frequency of urge urinary incontinence and improve urodynamic parameters (MCC and MDP) in patients with NDO at 6 weeks after treatment. This meta-analysis indicates that onabotulinumtoxinA is effective and safe for treating patients with NDO compared to placebo. Additionally, we did not observe any statistical or clinical differences in efficacy between 300 and 200 U dosages.

## Introduction

The neurogenic bladder is described as a bladder urethra dysfunction associated with central or peripheral nervous system diseases, such as spinal cord injury (SCI) and multiple sclerosis (MS) [1]. Patients with neurogenic bladder have lower urinary tract symptoms (LUTS), which can negatively affect the lives of adult patients. Overactive bladder (OAB) includes detrusor instability and detrusor hyperreflexia, which is described as neurogenic detrusor overactivity (NDO) in neurogenic bladder [2]. NDO is indicated by a combination of urinary frequency, urgency, and urgency urinary incontinence (UI) [3]. The urodynamic parameters of patients with NDO include high transient bladder pressures, low bladder capacity, and increasing UI episodes [4]. NDO seriously affects the physical and mental conditions of patients. Non-surgical treatment for NDO primarily includes oral anticholinergic drugs frequently combined with clean intermittent catheterization (CIC), which is considered first-line therapy for UI in these patients. However, many patients report a lower tolerance because of adverse events (AEs) resulting from the long-term use of anticholinergic drugs, which cause many complications, such as urinary tract infections (UTIs), urinary retention, hematuria, muscle weakness, depression, dry mouth, etc. [5].

Botulinum toxin (BTX) was first described by van Ermengem in 1897 and is considered the most potent and useful biological toxin for humans. BTX can control the activity of the detrusor by inhibiting the release of ACh at the neuromuscular junction. Smith and colleagues measured the biological activities of BTX using an animal model [6]. OnabotulinumtoxinA is an effective and safe therapy option in patients with NDO [7]. It was first approved by the Food and Drug Administration (FDA) in 2011 for the treatment of NDO. More recently, intradetrusor onabotulinumtoxinA has displayed significant benefits on UI, urodynamics, and treatment satisfaction compared to placebo, with fewer AEs [8]. Similarly, Chancellor and colleagues also reported the successful treatment of patients with NDO [9].

The clinical efficacy and safety of onabotulinumtoxinA treatment for NDO is dose related. Although previous studies have reported the efficacy and safety of intradetrusor injection of onabotulinumtoxinA for the treatment of NDO [9–11], studies comparing the efficacy of intradetrusor injections of different doses of onabotulinumtoxinA in patients are lacking [12–14]. In our study, we evaluated the efficacy of different doses of onabotulinumtoxinA for treating patients with NDO and the results could provide an important reference for the selection of the best dosage in clinical therapy. Moreover, we performed a network meta-analysis to summarize data from all included randomized controlled trials (RCTs) to assess the AEs associated with onabotulinumtoxinA use.

## Materials and Methods

### Search strategy

Two authors searched the PubMed, EMBASE and Controlled Trials Register databases for relevant English-language articles of clinical studies evaluating the efficacy and safety (or both) of intradetrusor injections of onabotulinumtoxinA published until October 1, 2015. The searches were limited to human subjects and RCTs. Patients with NDO who were refractory to oral antimuscarinic drugs for at least 3 months or those who had discontinued medical therapy because of side effects were included in this study. To reduce substantial bias, we selected the results that were reasonably obtained using professional document management tools, and we deleted duplicate published articles. The search keywords were as follows: botulinum toxin, onabotulinumtoxinA, neurogenic detrusor overactivity, randomized controlled trials, efficacy, and safety.

### Inclusion criteria and trial selection

Studies were selected for the meta-analysis if the following inclusion criteria were met: (a) population comprised of adult patients with neurogenic bladder disease, including SCI and MS; (b) intervention consisting of NDO with onabotulinumtoxinA; (c) comparison between BTX and placebo; (d) outcome of UI episodes per week, MCC, MDP, and the rate of the main frequent AEs, including UTs, urinary retention, hematuria, and muscle weakness; and (e) RCT study design. References meeting the inclusion criteria were evaluated by two reviewers independently, and disagreements were resolved by consensus with a third reviewer.

### Data collection

We carefully reviewed the titles, abstracts, and full texts of the articles. The data collection was performed independently by two authors using pre-designed data extraction forms. If the eligible articles reported insufficient data, we contacted the original authors and asked for the research details and digital data. For continuous data, summary estimates for each group (means and changes in means) with measures of variability (standard difference [SD] and 95% confidence interval [CI]) were extracted. The following information was collected from each study: (1) participants: number of patients, gender, neurogenic disorders, withdrawal, and/or loss to follow-up; (2) method: study design, randomized methods, allocation concealment method, blinding method, and other bias factors; (3) intervention: details of onabotulinumtoxinA treatment, such as the dose and treatment duration; (4) outcome: the mean number of UI episodes per week, the MCC, the MDP, and the reported rate of AEs, primarily including UTIs, urinary retention, hematuria, and muscle weakness; and (5) other: first author, country, and year of publication. Any disagreements were resolved by discussion and consensus.

### Quality assessment of the evidence

**Fig 1:**
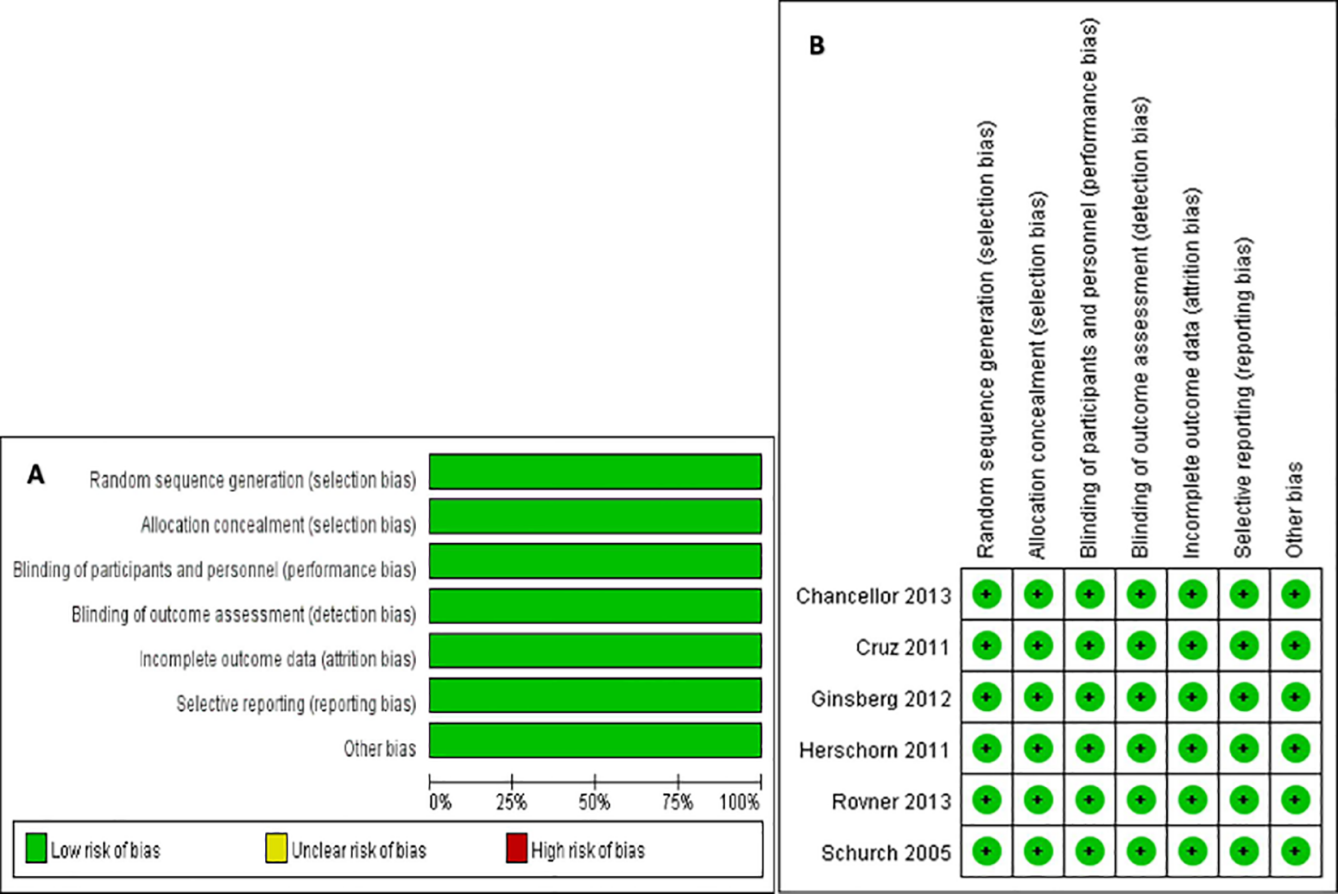
Results of the Risk of Bias Assessment. (A) The risk of bias graph. (B) The risk of bias summary.

Two reviewers assessed the methodological quality of the included studies. The quality of the retrieved RCTs included the assessment of random sequence generation, allocation concealment, blinding, incomplete outcome data, selective reporting, and other biases. In the Cochrane Collaboration Reviewers’ Handbook for Systemic Reviews of Interventions, for each item based on the question, the judgment (‘low risk’ of bias, ‘unclear risk’ of bias, or ‘high risk’ of bias;) is followed by a text box, which provides a basis for the description of the design, implementation, or measurement as a judgment. Based on the quality assessment criteria, each study was rated and assigned to one of the three following quality categories: A, all quality criteria were adequately met; B, one or more quality criteria were only partially met or were unclear; or C, one or more of the criteria were not included. The results of the risk of bias assessment are summarized in Fig 1. The level of evidence in this study is shown in Table 1.

**Table 1:** Characteristics of the Trials Included in this Study.

First author	Year	Country	No. of patients	% males	Loss to follow-up (%)	Neurogenic disorders	Study design	T/C	Active treatment mean follow-up, wks	Level of evidence
Chancellor	2013	USA, UK	416	41.1	0	MS: n = 227,SCI: n = 189	Randomized, double-blind, placebo-controlled	200 U: n = 135,300 U: n = 132,Placebo: n = 149	6 w, 12 w	A
Cruz	2011	Europe, North America, South Africa	275	43.6	1.4	MS: n = 154,SCI: n = 121	Randomized,double-blind, placebo-controlled	200 U: n = 92,300 U: n = 91,Placebo: n = 92	2, 6, 12 and 52 w	A
Herschorn	2011	Canada	58	58.6	10	MS: n = 38,SCI: n = 19	Randomized, double-blind, placebo-controlled	300 U: n = 28,Placebo: n = 29	1, 3, 4, 6, 24 and 36 w	A
Ginsberg	2012	Europe, USA	416	41.1	2.2	MS: n = 227,SCI: n = 189	Randomized, double-blind, placebo-controlled	200 U: n = 135,300 U: n = 132,Placebo: n = 149	6 w, 12 w	A
Rovner	2013	USA	691	42.1	2	MS: n = 381,SCI: n = 310	Randomized, double-blind, placebo-controlled	200 U: n = 241,300 U: n = 227,Placebo: n = 223	2, 6, 12 and 52 w	A
Schurch	2005	Belgium, France	59	36	0	MS: n = 53,SCI: n = 6	Randomized, double-blind, placebo-controlled	200 U: n = 19,300 U: n = 19,Placebo: n = 21	2, 6, 12, 18 and 24 w	A

MS: multiple sclerosis; SCI: spinal cord injury; T/C: treatment/control.

### Statistical analysis

Differences are expressed as RRs with 95% CIs for dichotomous outcomes and the mean difference (MD) with 95% CIs for continuous outcomes. Heterogeneity across studies was tested using the *I*^*2*^ statistic. Studies with an *I*^*2*^ statistic = 0 were considered to have no heterogeneity. Larger *I*^*2*^ values indicated greater heterogeneity. Studies with an *I*^*2*^ statistic >50% were considered to have significant heterogeneity. A fixed-effects model was used if there was no significant heterogeneity (*I*^*2*^<50%). Otherwise, the meta-analysis was performed using the random-effects model to further explore the potential sources of heterogeneity. We used the change in the mean (SD) for the meta-analysis, which reduces the risk of bias. The publication bias was evaluated using the Cochrane Collaboration Reviewers’ Handbook for Systemic Reviews of Interventions v.5.1.0, and all of the statistical analyses were performed using the same software.

## Results

### Study selection and characteristics of the individual studies

**Fig 2:**
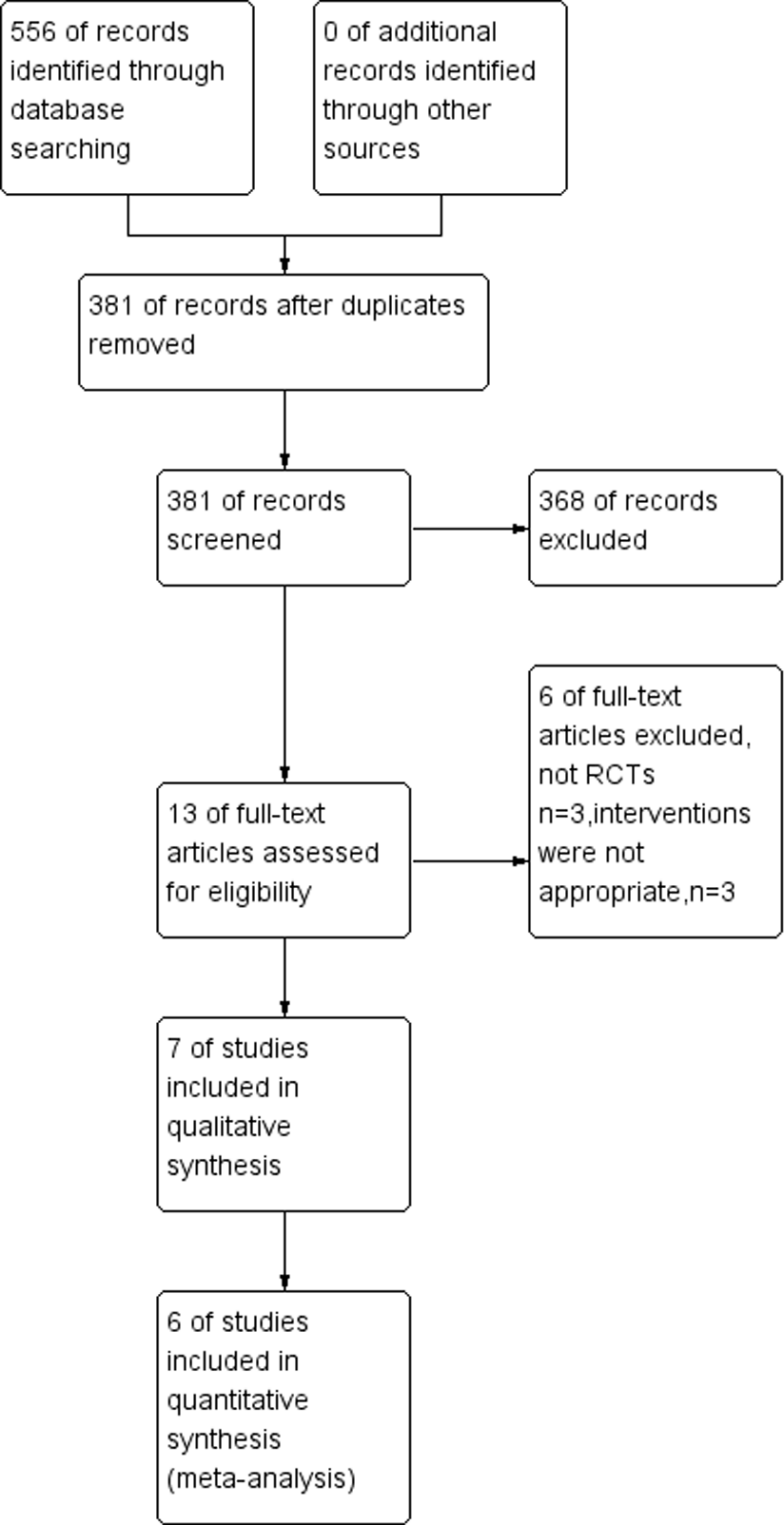
Flow Diagram of the Study Selection Process.

A total of 556 studies were identified by the initial database search. Of these, 175 articles were excluded based on the titles and abstracts. A total of 366 articles were excluded based on the inclusion criteria after reading the full text. The remaining thirteen full-text articles were reviewed for more detailed evaluation, and seven were also excluded because they were not RCTs or the interventions were inappropriate. Finally, six RCTs that met our inclusion criteria were included in this meta-analysis. The selection process for the RCTs included is shown in Fig 2. The baseline characteristics of the studies included in our meta-analysis are listed in Table 1.

### Quality of the individual studies

The six RCTs were randomized, double-blind, placebo controlled trials enrolling a total of 1,915 patients. The results of the risk of bias assessment are summarized in Fig 1. We found that the quality of those RCT studies was level A (Table 1).

### Efficacy

Efficacy analyses were performed on the intent-to-treat population (all randomized patients). The primary efficacy measure was the change from baseline for the mean number of weekly UI episodes. The primary point of assessment in each cycle was week 6 after each treatment, identical to the end point in the pivotal studies. Additional efficacy variables at week 6 included changes in the MCC and MDP.

### Frequency of urinary incontinence episodes

**Fig 3:**
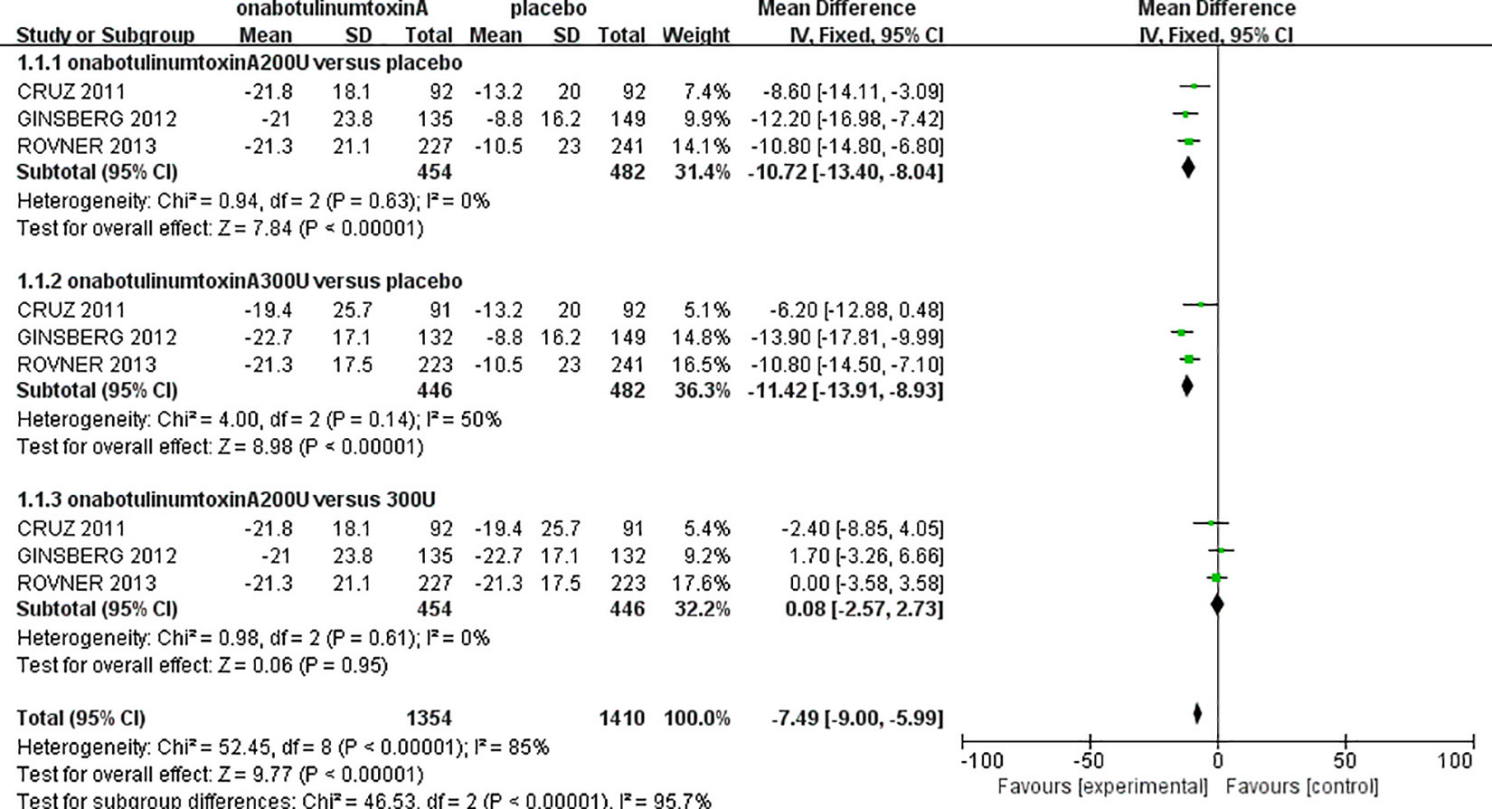
Forest Plot of the Changes of Urinary Incontinence Episodes per Week (at 6 Weeks).

Approximately 1,382 patients in three RCT studies [11–13], including the onabotulinumtoxinA200U group (n = 454), the onabotulinumtoxinA300U group (n = 446), and the placebo group (n = 482), reported data for the mean change from baseline of UI episodes per week (at 6 weeks) (Fig 3). There were significant decreases in UI episodes in the onabotulinumtoxinA200U group (MD: -10.72, 95% CI: -13.4 to -8.04, P<0.00001) and the 300U group (MD: -11.42, 95% CI: -13.91 to -8.93, P<0.00001). Compared to the placebo group, there was no significant heterogeneity in either onabotulinumtoxinA group. Interestingly, there were no significant differences between the onabotulinumtoxinA-treated groups in the number of weekly UI episodes (MD: 0.08, 95% CI: -2.57 to 2.73, P = 0.95). This result suggests that onabotulinumtoxinA has significant beneficial effects in UI episodes compared to a placebo.

### Maximum cystometric capacity and maximum detrusor pressure

**Fig 4:**
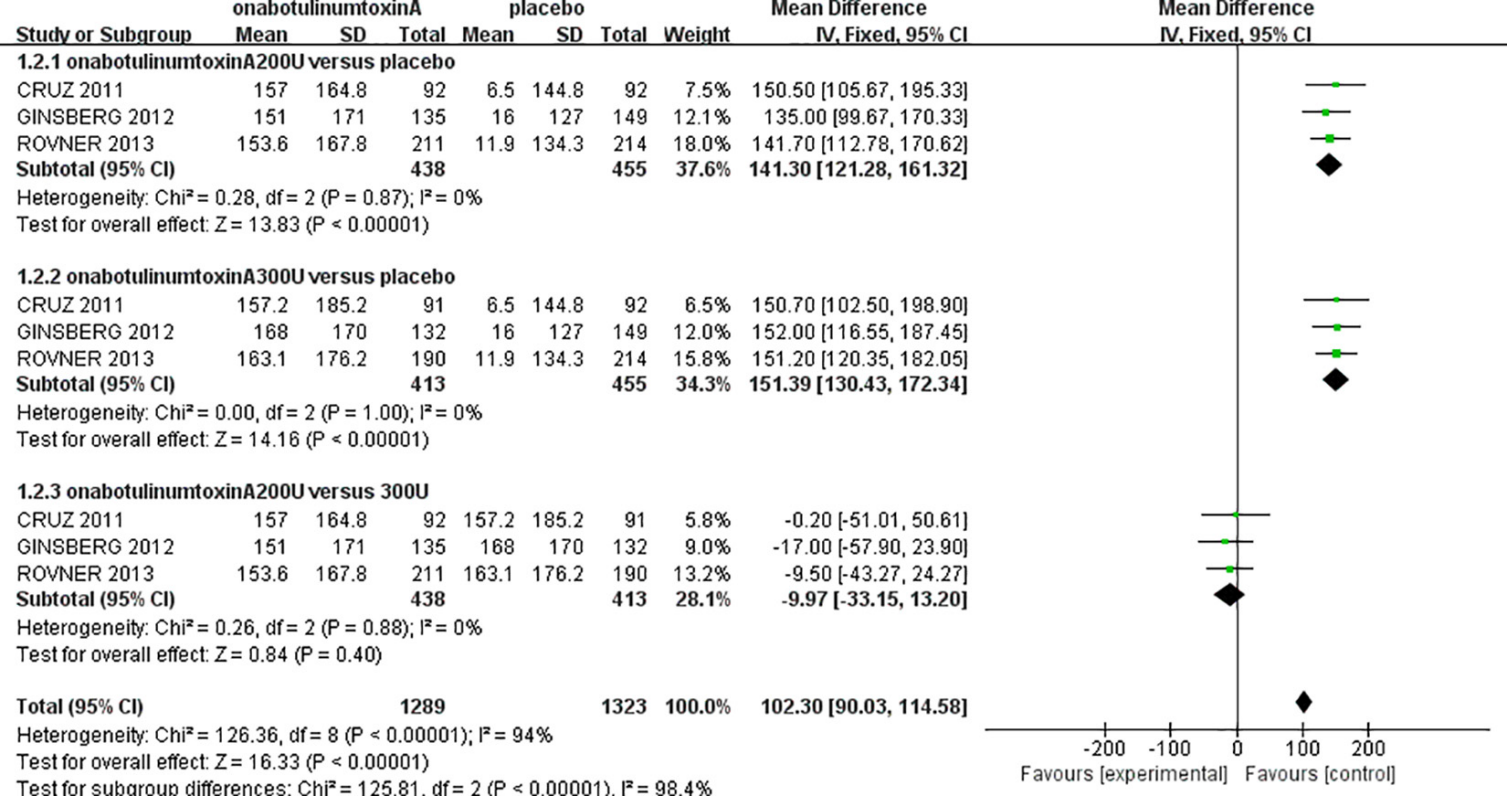
Forest Plot of the Maximum Cystometric Capacity (MCC) (at 6 Weeks).

**Fig 5:**
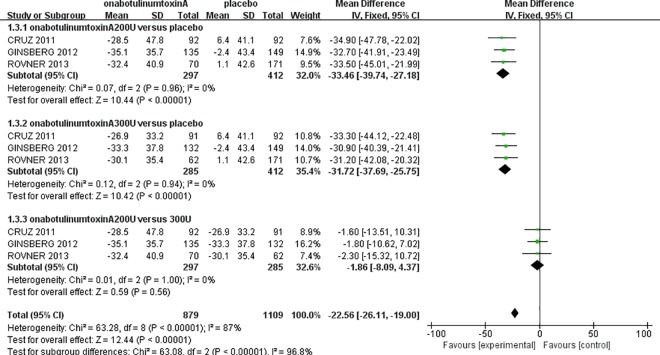
Forest Plot of the Maximum Detrusor Pressure (MDP) (at 6 Weeks).

The urodynamic parameters in this meta-analysis showed improvements comparable to those reported previously. The three randomized controlled studies [11–13] showed that onabotulinumtoxinA was significantly superior for increasing the MCC and reducing the MDP compared to the placebo. For MCC, there was no heterogeneity in either onabotulinumtoxinA-treated group (onabotulinumtoxinA200U: *I*^*2*^ = 0%, P = 0.87; onabotulinumtoxinA300U: *I*^*2*^ = 0%, P = 1.00); therefore, the fixed-effect model was used. The analysis demonstrated that the MCC in the onabotulinumtoxinA-treated groups was larger than in the placebo group (onabotulinumtoxinA200U: MD: 141.30, 95% CI: 121.28 to 161.32, P<0.00001; onabotulinumtoxinA300U: MD: 151.39, 95% CI: 130.43 to 172.34, P<0.00001) (Fig 4). However, there was no significant difference between the onabotulinumtoxinA200U and 300U groups (MD: -9.97, 95% CI: -33.15 to 13.20, P = 0.40). There was also no heterogeneity for the MDP in either onabotulinumtoxinA-treated group compared to the placebo (onabotulinumtoxinA200U: *I*^*2*^ = 0%, P = 0.96; 300 U: *I*^*2*^ = 0%, P = 0.94). The MDP result showed that there was a significant reduction in the onabotulinumtoxinA-treated groups (onabotulinumtoxinA200U: MD: -33.46, 95% CI: -39.74 to -27.18, P<0.00001; 300 U: MD: -31.72, 95% CI: -37.69 to -25.75, P<0.00001). We also observed that there was no significant difference between the two onabotulinumtoxinA-treated groups (MD: -1.86, 95% CI: -8.09 to 4.37, P = 0.56) (Fig 5).

### Safety

All of the studies that met the inclusive selection criteria in this meta-analysis reported an excellent safety profile for onabotulinumtoxinA intradetrusor injection. Five studies [8,10–13] including 1,473 patients (951 in the onabotulinumtoxinA group and 522 in the placebo group) reported that the AEs were either transient or easily manageable, including the rate of UTIs, urinary retention, hematuria, and muscle weakness. Any AEs that occurred as a result of the injection procedure were not included in this meta-analysis. Urologic events (UTIs, urinary retention, and hematuria) were dose-related in patients not using CIC at baseline. These events were likely treatment related. Based on our meta-analysis, there was no heterogeneity in these RCTs (Table 2). The results report the main AEs as UTIs (RR: 1.47, 95% CI: 1.29 to 1.67, P<0.00001), urinary retention (RR: 5.58, 95% CI: 3.53 to 8.83, P<0.00001), hematuria (RR: 1.70, 95% CI: 1.01 to 2.85, P = 0.05), and muscle weakness (RR: 2.59, 95% CI: 1.36 to 4.91, P = 0.004). Data from the trials indicated that the use of onabotulinumtoxinA for patients who have detrusor overactivity leads to greater relative complications than a placebo.

**Table 2:** Analysis Outcomes of Adverse Events (Onabotulinumtoxina versus Placebo).

Adverse events	Number of RCTs	Heterogeneity (*I*^*2*^)	RR	95% CI	P Value
urinary tract infections	5	0%	1.47	1.29–1.67	<0.00001
urinary retention	5	28%	5.58	3.53–8.83	<0.00001
hematuria	5	0%	1.70	1.01–2.85	0.05
muscle weakness	5	0%	2.59	1.36–4.91	0.004

RR: Relative risk; CI: Confidence interval; RCTs: randomized controlled trials.

## Discussion

NDO is a widespread chronic illness that impairs millions of people worldwide. Many patients seek an appropriate therapeutic option that provides durable treatment responses with the least AEs. The first-line treatment for NDO includes antimuscarinics and catheterization, preferably CIC [15]. However, the treatment effect is often unsatisfactory; therefore, we must consider other options. OnabotulinumtoxinA has been demonstrated to be a good therapeutic method for patients with NDO, and the use of onabotulinumtoxinA may improve urinary symptoms and quality of life (QoL) [16].

We performed a meta-analysis with a larger sample size to evaluate the efficacy and safety of onabotulinumtoxinA for NDO in adult patients. In each onabotulinumtoxinA-treated group, we observed a significant increase in MCC and a significant decrease in MDP compared to the placebo group (Figs 4 and 5). Comparing the mean number of UI episodes per week before and after treatment in NDO patients revealed a significant decrease in UI episodes after treatment with onabotulinumtoxinA (Fig 3). Our meta-analysis demonstrated a statistically significant improvement in the frequency of incontinence and urodynamic parameters. Oral antimuscarinics before onabotulinumtoxinA injection has a major effect on the results. However, only two studies [11,12] indicated that patients treated with onabotulinumtoxinA could use antimuscarinics; therefore, the potential effect on the efficacy of onabotulinumtoxinA cannot be definitively evaluated. Moreover, Ehren and colleagues reported that almost all patient QoLs were significantly improved in the onabotulinumtoxinA-treated groups following treatment, and changes in the urodynamic parameters were accompanied by improvements in patient symptoms [17].

Although the effectiveness of onabotulinumtoxinA for the treatment of NDO has been verified, the dose used in clinical practice remains controversial. Nuanthaisong U and colleagues reported that in patients with neurogenic bladder, over 360 U of onabotulinumtoxinA was an effective treatment [18]. However, our review mainly focused on 200 or 300 U. In this study, the intradetrusor injection of onabotulinumtoxinA (300 or 200 U) had a significant therapeutic effect on NDO patients who were refractory to treatment with oral antimuscarinic drugs. Although the limited number of patients in this study set prevented us from drawing definite conclusions about efficacy and safety, our meta-analysis added new RCT articles based on the previous systematic review. Considering the discussed results, our study confirmed the results of previous studies regarding the beneficial effects of intradetrusor injection of onabotulinumtoxinA in patients with NDO.

Both the previous systematic review and our meta-analysis showed that the incidence of the main frequent AEs, including UTIs, urinary retention, and hematuria, was slightly higher in the onabotulinumtoxinA-treated groups [8,10–13]. Except for the AEs of the uropoietic system, there were no other AEs, such as muscle weakness, to assess in the previous study. Muscle weakness is another important complication after onabotulinumtoxinA injection that is rarely observed by patients and physicians. In our network meta-analysis, we also showed a clinically relevant difference in muscle weakness (RR: 2.59, 95% CI: 1.36 to 4.91, P = 0.004) (Table 2), which is another main finding.

The efficacy and safety of onabotulinumtoxinA injection for the treatment of NDO has been shown in different studies [19–23]. However, there are a limited number of studies comparing the effects of different doses of onabotulinumtoxinA injection in NDO. Mangera and colleagues conducted a systematic review of the role of onabotulinumtoxinA for the management of lower urinary tract disease. They also concluded that the effective dose of onabotulinumtoxinA was different when treating patients with NDO [24]. To our knowledge, the highlight of our systematic review is the assessment of different onabotulinumtoxinA doses on the clinical effect of treating NDO patients. However, for UI episodes and urodynamic parameters in our study, the improvements in MDP and MCC in the onabotulinumtoxinA200U and 300U groups versus the placebo were statistically significant, and for both groups, the changes were not significant. It is unknown whether higher or lower doses are more effective for patients who fail to gain a specific benefit with 200 and 300 U, which may guide future research on the topic.

### Study limitations

Several potential limitations should be considered in this meta-analysis. First, almost all of the included studies were RCTs, and the few studies not clearly specifying any information about the patients were excluded. However, only three RCTs were categorized for analysis, and these limited trials with insufficient data may influence the conclusion. Second, the small sample size, including patients without adequate information or without detailed test results, may increase the risk of bias assessment. Third, the articles of our meta-analysis primarily used onabotulinumtoxinA200U or 300U for NDO, although other doses are used to treat NDO and are also safe and efficacious. These were not included in this study; therefore, future high quality prospective RCTs are required to determine the optimal dose, injection technique, favorable timing, and depth of the injection for onabotulinumtoxinA therapy. Fourth, repeated injections are necessary to maintain a sustained therapeutic effect. Until now, there have been only a few studies evaluating the long-term effects of repeated onabotulinumtoxinA injection on bladder function [11,13,24]. The effect of onabotulinumtoxinA treatment on clinical outcomes, urodynamic parameters, and QoL was studied for repeated onabotulinumtoxinA injections [25]. Therefore, within the limits of this study, patients with NDO had an acceptable response after repeated injections for more than 3 months, and data on the long-term efficacy and safety of onabotulinumtoxinA were insufficient. Furthermore, high-quality trials with strict inclusion criteria conducted worldwide are proposed to learn more about the efficacy and safety of the therapies for NDO.

## Conclusions

We conducted a network meta-analysis, which assessed all available information from clinical RCTs. The result indicates that the use of onabotulinumtoxinA in NDO is a well-established treatment method with good outcomes and minimal AEs. In addition, we did not observe any significant differences in efficacy between the 300 U dose and the 200 U dose.

## Supporting Information

S1 File PRISMA 2009 flow diagram. (PDF)

S2 File PRISMA 2009 checklist. (PDF)
