# Supplementary material for: Efficacy and Safety of OnabotulinumtoxinA in Patients with Neurogenic Detrusor Overactivity: A Systematic Review and Meta-Analysis of Randomized Controlled Trials
Source: PLoS One. 2016 Jul 27;11(7):e0159307. doi: 10.1371/journal.pone.0159307 (PMC4963110; doi:10.1371/journal.pone.0159307)
Supplement: S1 File — (PDF) [file pone.0159307.s001.pdf]

PRISMA 2009 Flow Diagram

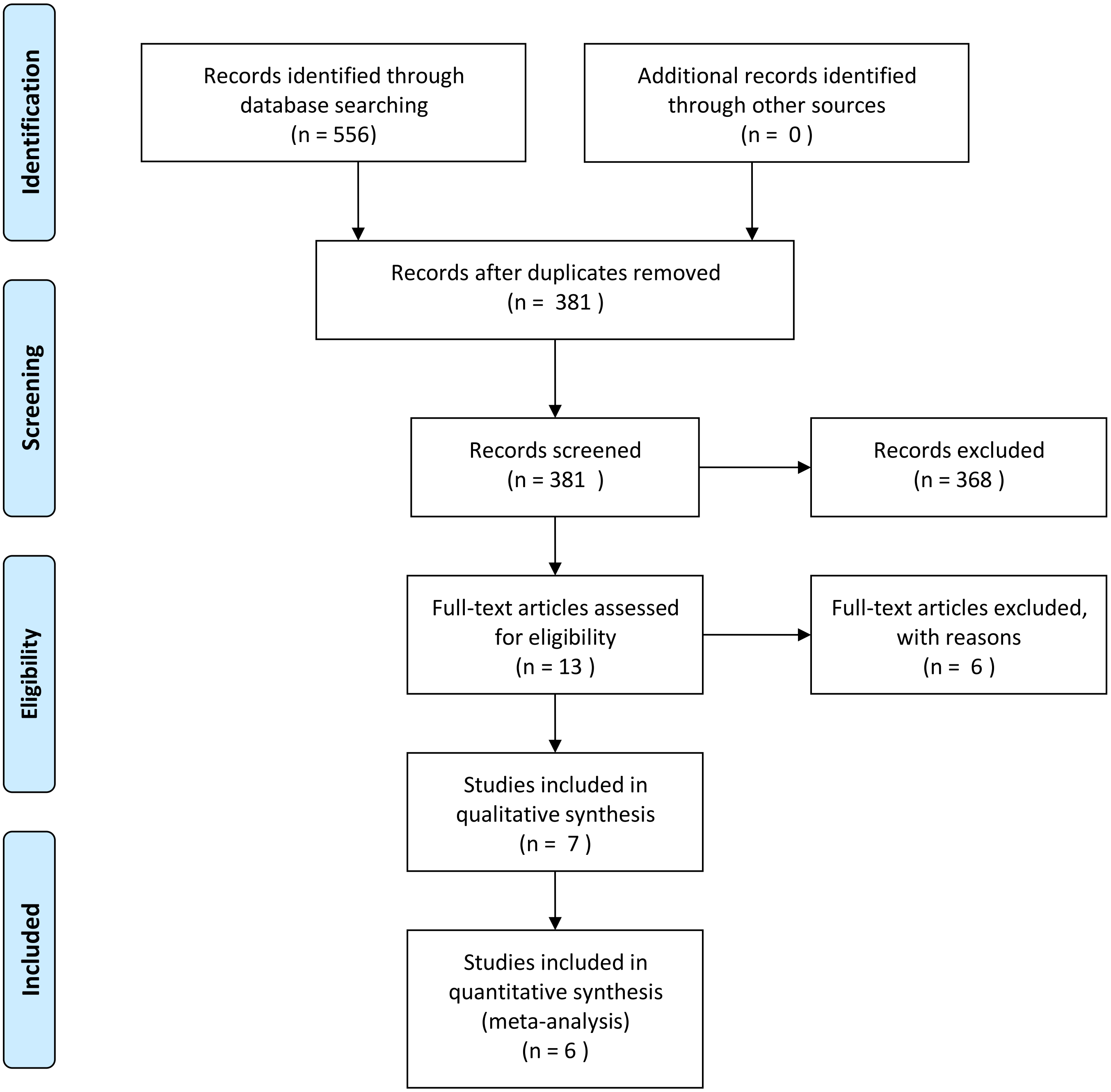

From: Moher D, Liberati A, Tetzlaff J, Altman DG, The PRISMA Group (2009). *P*referred *R*eporting *I*tems for *S*ystematic Reviews and *M*eta-*A*nalyses: The PRISMA Statement. PLoS Med 6(7): e1000097. doi:10.1371/journal.pmed1000097

For more information, visit www.prisma-statement.org.
