# Supplementary material for: Efficacy and Safety of OnabotulinumtoxinA in Patients with Neurogenic Detrusor Overactivity: A Systematic Review and Meta-Analysis of Randomized Controlled Trials
Source: PLoS One. 2016 Jul 27;11(7):e0159307. doi: 10.1371/journal.pone.0159307 (PMC4963110; doi:10.1371/journal.pone.0159307)
Supplement: S2 File — (PDF) [file pone.0159307.s002.pdf]

PRISMA 2009 Checklist

Section/topic	#	Checklist item	Reported on page #
**TITLE**			
Title	1	Identify the report as a systematic review, meta-analysis, or both.	meta-analysis
**ABSTRACT**			
Structured summary	2	Provide a structured summary including, as applicable: background; objectives; data sources; study eligibility criteria, participants, and interventions; study appraisal and synthesis methods; results; limitations; conclusions and implications of key findings; systematic review registration number.	Abstract
**INTRODUCTION**			
Rationale	3	Describe the rationale for the review in the context of what is already known.	
Objectives	4	Provide an explicit statement of questions being addressed with reference to participants, interventions, comparisons, outcomes, and study design (PICOS).	(a)Participants:adult critically ill patients who have neurogenic bladder diease; (b)intervention: Neurogenic Detrusor Overactivity with onabotulinumtoxinA; (c)comparison: Neurogenic Detrusor Overactivity with placebo; (d) outcome: UI episodes per week, maximum cystometric capacity (MCC), maximum detrusor pressure (MDP) and the rate of mainly frequent adverse events(including urinary tract infections , urinary retention , hematuria and muscle weakness); (e) design: RCTs.
**METHODS**			
Protocol and registration	5	Indicate if a review protocol exists, if and where it can be accessed (e.g., Web address), and, if available, provide registration information including registration number.	
Eligibility criteria	6	Specify study characteristics (e.g., PICOS, length of follow-up) and report characteristics (e.g., years considered, language, publication status) used as criteria for eligibility, giving rationale.	Inclusion criteria and trials selection
Information sources	7	Describe all information sources (e.g., databases with dates of coverage, contact with study authors to identify additional studies) in the search and date last searched.	Two authors searched the PubMed, EMBASE and the Controlled Trials Register databases for relevant English-language articles that concerned clinical studies evaluating the efficacy and safety (or both) of intradetrusor injection of onabotulinumtoxinA in

PRISMA 2009 Checklist

Section/topic	#	Checklist item	Reported on page #
			adults published up to October 1, 2015.
Search	8	Present full electronic search strategy for at least one database, including any limits used, such that it could be repeated.	Search strategy (botulinum toxin[Title/Abstract]) AND Neurogenic Detrusor Overactivity [Title/Abstract]
Study selection	9	State the process for selecting studies (i.e., screening, eligibility, included in systematic review, and, if applicable, included in the meta-analysis).	Inclusion criteria and trials selection
Data collection process	10	Describe method of data extraction from reports (e.g., piloted forms, independently, in duplicate) and any processes for obtaining and confirming data from investigators.	Data collection
Data items	11	List and define all variables for which data were sought (e.g., PICOS, funding sources) and any assumptions and simplifications made.	Table 1-Characteristics of trails included in this study
Risk of bias in individual studies	12	Describe methods used for assessing risk of bias of individual studies (including specification of whether this was done at the study or outcome level), and how this information is to be used in any data synthesis.	The quality of retrieved RCTs included assessment of random sequence generation, allocation concealment, blinding, incomplete outcome data, selective reporting and other bias.<br>Figure1-All results of the risk of bias assessment
Summary measures	13	State the principal summary measures (e.g., risk ratio, difference in means).	Differences were expressed as RRs with 95% CI for dichotomous outcomes, and mean difference (MD) with 95% CIs for continuous outcomes.
Synthesis of results	14	Describe the methods of handling data and combining results of studies, if done, including measures of consistency (e.g., I^2) for each meta-analysis.	Heterogeneity across studies was tested by using the I^2 statistic. Studies with an I^2 statistic of =0 were considered to have no heterogeneity. The larger the value of I^2 statistic, the higher the heterogeneity may be. Studies with an I^2 statistic of <50% were considered to have low heterogeneity, those with an I^2 statistic of 50% to 75% were considered to have moderate heterogeneity, and those with an I^2 statistic of >75% were considered to have high heterogeneity.

PRISMA 2009 Checklist

Risk of bias across studies	15	Specify any assessment of risk of bias that may affect the cumulative evidence (e.g., publication bias, selective reporting within studies).	Quality assessment of the evidence
Additional analyses	16	Describe methods of additional analyses (e.g., sensitivity or subgroup analyses, meta-regression), if done, indicating which were pre-specified.	
**RESULTS**			
Study selection	17	Give numbers of studies screened, assessed for eligibility, and included in the review, with reasons for exclusions at each stage, ideally with a flow diagram.	Figure 2-A flow diagram of the study selection process
Study characteristics	18	For each study, present characteristics for which data were extracted (e.g., study size, PICOS, follow-up period) and provide the citations.	Study selection and characteristics of the individual studies
Risk of bias within studies	19	Present data on risk of bias of each study and, if available, any outcome level assessment (see item 12).	The results of the risk of bias assessment are summarized in Figure 1. We found the level of quality of those RCTs studies were A (Table-1).
Results of individual studies	20	For all outcomes considered (benefits or harms), present, for each study: (a) simple summary data for each intervention group (b) effect estimates and confidence intervals, ideally with a forest plot.	Table 1-Characteristics of trails included in this study
Synthesis of results	21	Present results of each meta-analysis done, including confidence intervals and measures of consistency.	The frequency of urinary incontinence episodes(Figure3). Maximum cystometric capacity (MCC) and Maximum detrusor pressure (MDP)(Figure4, Figure5)
Risk of bias across studies	22	Present results of any assessment of risk of bias across studies (see Item 15).	The results of the risk of bias assessment are summarized in Figure 1
Additional analysis	23	Give results of additional analyses, if done (e.g., sensitivity or subgroup analyses, meta-regression [see Item 16]).	
**DISCUSSION**			
Summary of evidence	24	Summarize the main findings including the strength of evidence for each main outcome; consider their relevance to key groups (e.g., healthcare providers, users, and policy makers).	The highlight of our systematic review is the assessment of different onabotulinumtoxin A doses on clinical effect in treating NDO patients. There was no significantly difference between onabotulinumtoxin

PRISMA 2009 Checklist

			A200U and 300U.
Limitations	25	Discuss limitations at study and outcome level (e.g., risk of bias), and at review-level (e.g., incomplete retrieval of identified research, reporting bias).	Study limitions
Conclusions	26	Provide a general interpretation of the results in the context of other evidence, and implications for future research.	Conclusions
**FUNDING**			
Funding	27	Describe sources of funding for the systematic review and other support (e.g., supply of data); role of funders for the systematic review.	The outstanding young leader funded projects of Colleges and Universities in Shanxi Province (JINJIAOKE[2012] 10)

From: Moher D, Liberati A, Tetzlaff J, Altman DG, The PRISMA Group (2009). Preferred Reporting Items for Systematic Reviews and Meta-Analyses: The PRISMA Statement. PLoS Med 6(7): e1000097. doi:10.1371/journal.pmed1000097

For more information, visit: **www.prisma-statement.org**.
